# How do patients interpret and respond to a single-item global indicator of cancer treatment tolerability?

**DOI:** 10.1007/s00520-022-07484-7

**Published:** 2022-12-16

**Authors:** John Devin Peipert, Sara Shaunfield, Karen Kaiser, Patricia I. Moreno, Rina S. Fox, Sheetal Kircher, Nisha Mohindra, Edward Ip, Fengmin Zhao, Lynne Wagner, David Cella

**Affiliations:** 1 Department of Medical Social Sciences, Northwestern University Feinberg School of Medicine, 625 Michigan Ave, 21st Floor, IL 60611 Chicago, USA; 2 Department of Public Health Sciences, University of Miami Miller School of Medicine, Miami, FL USA; 3 University of Arizona College of Nursing, Tucson, AZ USA; 4 University of Arizona Cancer Center, Tucson, AZ USA; 5 Division of Hematology and Oncology, Department of Medicine, Northwestern University Feinberg School of Medicine and the Robert H. Lurie Comprehensive Cancer Center, IL Chicago, USA; 6 Department of Biostatistics and Data Science, Wake Forest School of Medicine, Winston-Salem, NC USA; 7 ECOG-ACRIN Biostatistics Center, Dana-Farber Cancer Institute, Boston, MA USA; 8 Department of Biostatistics, Harvard T. H. Chan School of Public Health, Boston, MA USA; 9 Department of Social Sciences and Health Policy, Wake Forest School of Medicine, Winston-Salem, NC USA

**Keywords:** Treatment tolerability, Clinical trials, FACT

## Abstract

**Background:**

There is increasing interest in patient-reported measures of cancer treatment tolerability. A global measure of bother, the FACT GP5 item (“I am bothered by side effects of treatment”) is potentially useful for regulatory, research, and clinical use. To understand this item’s appropriateness for capturing treatment tolerability, we conducted cognitive interviews on this item with 3 samples of cancer patients.

**Methods:**

Patients with ovarian cancer (Study 1: *N* = 21; on treatment), lymphoma (Study 2: *N* = 14; on treatment), and colorectal or lung cancer (Study 3: *N* = 16; treatment naïve) were interviewed about GP5’s understandability and relevance to their treatment side effects. What patients think about when answering GP5 was also assessed. In all studies, the interview included both structured and open-ended questions. Qualitative data were coded to extract themes and responses to structured questions were tallied.

**Results:**

Most patients on treatment (Studies 1 and 2) reported that the GP5 item wording is appropriate (88%) and its meaning is clear (97%). They were very confident or confident in their response (97%) and stated that GP5 was relevant to their cancer experience (97%). When answering GP5, patients considered their treatment and specific side effects. A large proportion (40%) of the treatment-naïve (Study 3) patients reported that GP5 was not relevant to their cancer treatment, and the largest proportion responded to GP5 thinking of negative side effect expectancies.

**Conclusion:**

This study provides assurance that GP5 is a useful indicator of treatment tolerability, and is meaningful to people with cancer, especially once they have started treatment.

## Introduction

Tolerability in cancer clinical trials has traditionally focused on the degree to which overt adverse effects of treatment can be tolerated by patients and has typically been assessed by clinician report using the Common Terminology Criteria for Adverse Events (CTCAE) and other trial data (e.g., dose modifications, dose discontinuations, hospitalizations) [1]. However, patients often experience side effects that are not detected by clinicians but have a significant impact on their daily activities and quality of life [2, 3]. Patient reports of adverse events (e.g., pain, nausea, fatigue) are generally earlier and more frequent than clinician reports of the same adverse events [3]. Furthermore, patient reports of symptomatic adverse events are more strongly associated with overall health status, while clinician reports predict clinical events like emergency room visits and death. Therefore, there is increasing interest in incorporating patient-reported measures of tolerability in cancer clinical trials to understand the extent to which symptomatic adverse events affect patients’ ability or desire to adhere to cancer therapies [4–6]. In line with this shift, a recent, patient-focused definition of tolerability has been offered: “The tolerability of a medical product is the degree to which symptomatic and non-symptomatic adverse events associated with the product’s administration affect the ability or desire of the patient to adhere to the dose or intensity of therapy. A complete understanding of tolerability should include direct measurement from the patient on how they are feeling and functioning while on treatment.” [4].

Measures that reflect this new patient-focused definition of tolerability are needed. The patient-reported outcome (PRO) version of the CTCAE was developed to assess specific symptomatic adverse events directly from the patient [7–9]. However, in addition to assessing patient-reported symptomatic adverse events, it is important to assess the global impact or bother associated with treatment side effects to capture the aggregate impact of multiple side effects and help compare across treatments with different side effect profiles [4]. The United States Food and Drug Administration (FDA) recently published its “Core Patient-Reported Outcomes in Cancer Clinical Trials” draft guidance in which it identified a set of key PROs, including those aimed at capturing tolerability [10]. Most directly, these include symptomatic adverse events and an overall side effect impact summary measure [10].

One of the leading options for an overall side effect impact summary measure is the Functional Assessment of Cancer Therapy‐General (FACT) item GP5: “I am bothered by side effects of treatment” [10]. The GP5 item was introduced as part of the FACT – General (FACT-G) measure, wherein it is included in the Physical Well-Being subscale [11]. The GP5 was included in the FACT-G’s item generation and review process whereby its importance to health-related quality of life (HRQoL) in cancer was established through input from advanced cancer patients and expert clinicians [11]. Since then, the GP5 has been included in numerous cancer clinical trials, often as part of the FACT-G or other measures in the FACT system. In addition to its inclusion in multi-item FACT scales, it has also been analyzed independently (as a single item) in cancer trials [12–14].

The GP5’s measure properties have been examined, providing support for its use independently. Previous research demonstrates that greater bother from cancer therapy side effects is associated with increased clinician‐reported adverse events, worse health-related quality of life, and a greater likelihood of discontinuing treatment prior to completing protocol therapy [15, 16]. Greater bother from side effects is also associated with lower quality of life and less enjoyment of life [15, 17], which may further reduce tolerability among patients undergoing cancer therapy. Though initial development of this item was informed by patient input, there is opportunity to expand on the original qualitative evaluation by elucidating what specifically patients think of when coming to a response for GP5, how they interpret the term “bother” in particular, and how this item is perceived by patients prior to starting systemic treatment, which is a common aspect of cancer trial design.

The goal of the current study was to assess the patient-centered comprehensibility and relevance of the FACT GP5 item. Using data from three studies, we examined how patients with various cancer diagnoses, including those naïve to cancer therapy, interpret and respond to this item. We were particularly interested in learning more about what experiences and symptom characteristics (e.g., severity, frequency) influence how patients rate their global bother related to cancer therapy side effects.

## Methods

### Study design and participants

Data were drawn from 3 separate qualitative studies. Two of the studies aimed to establish the content validity of 2 symptom index measures within the FACIT system: the NCCN/FACT Ovarian Cancer Symptom Index – 18 Item Version (NFOSI-18) and the NCCN/FACT Lymphoma Cancer Symptom Index – 18 Item Version (NFLymSI-18). The FACT GP5 item (I am bothered by side effects of treatment) is included in each of these instruments. Therefore, analysis of data from these studies (referred to as Studies 1 and 2 below) is on a secondary basis. The third study was specifically focused on understanding responses to GP5 among patients naïve to systemic cancer therapy. Accordingly, the data from this study (Study 3 below) is analyzed on a primary basis. In each study, patients were recruited from the Robert H. Lurie Comprehensive Cancer Center of Northwestern University.

#### Study 1: content validation of NFOSI-18 in advanced ovarian cancer [18]

The first study was a qualitative, cognitive interview study that aimed to assess the content validity of the NFOSI-18 [18]. In addition, it explored patient interpretations of bother (and whether it was related to severity), when responding to items using the term bother, such as GP5. After establishing eligibility, a convenience sample of patients were recruited for the study. Eligible patients were female; aged ≥ 18 years; diagnosed with stage III or IV high-grade serous adenocarcinoma of epithelial ovarian, fallopian tube, or primary peritoneal cancer; and assessed as Eastern Cooperative Oncology Group performance status rating (ECOG PSR) 0 to 2. In addition, patients must have been able and willing to sign an informed consent document. Patients not fluent in English were excluded. Sample size was based on evidence that saturation (point at which no new concepts arise) often occurs as early as 12 interviews [19] as well as considerations of measure complexity, population diversity [20, 21], and prior experience conducting cognitive interviews. The study was reviewed by the Northwestern University Institutional Review Board, which determined it was non-human subjects research (STU00203656). Informed consent was obtained for all study participants.

#### Study 2: patient-reported outcome dossier in support of the NFLymSI-18 indolent B cell non-Hodgkin’s lymphoma (iNHL) patients [22]

The second study was a qualitative, concept elicitation and cognitive interview study that aimed to evaluate the content validity of FLymSI-18 in patients with iNHL [22]. After establishing eligibility, a convenience sample of patients were recruited for the study. Eligible patients had histologically confirmed diagnosis of iNHL and had received one or more prior lines of treatment. Patients aged ≥ 18 years, with ECOG PSR ≤ 2, and life expectancy ≥ 3 months were eligible. Patients were recruited after being identified in the medical record and were then approached in clinic. Each participating patient gave consent. Patients were recruited until saturation was reached. Saturation was evaluated after 12 interviews, and every 3 interviews thereafter, if necessary, until saturation was obtained. Saturation was defined as 3 interviews occurring without new, relevant content introduced. The study was approved by the Northwestern University IRB (STU00102531) and informed consent was obtained for all study participants.

#### Study 3: GP5 cognitive interview study among treatment-naïve patients

The third study was a qualitative, cognitive interview study that explored understanding of the GP5 item among newly diagnosed patients who had not received systemic chemotherapy or radiation therapy for cancer. After establishing eligibility, a convenience sample of patients were recruited for the study. Eligible patients were aged ≥ 18 years, had a diagnosis of any cancer, and had never received systemic cancer therapy. Patients who had surgery for their cancer were permitted to participate and still considered naïve to systemic cancer treatment. We excluded patients unwilling to provide informed consent and those who were not fluent in English. Each patient gave oral consent to participate. Since the study was low risk and conducted by teleconference, oral consent was deemed by ethics reviewers to be appropriate. We recruited and consented patients until saturation was reached. Saturation was evaluated with the same procedure as described for Study 2. The Northwestern University IRB that reviewed this study protocol made a determination of non-human subjects exemption (STU00210027).

### Cognitive interview methods

For Studies 1 and 2, a trained interviewer approached eligible patients, explained the study, and obtained written informed consent. Interviews were conducted in person during the patient’s infusion appointment or in a private room following the patient’s clinic visit. Interviews were audio recorded. For Study 3, clinicians identified potentially eligible patients, and then trained study personnel contacted these patients by phone. If the patient was interested and willing to participate, an oral consent form was completed before conducting the interview.

The overall cognitive interview approach was similar in each study. Semi-structured cognitive interview guides were used to gather patient input. For each study, the cognitive interviewing protocol was based on the work of Willis [23] and ascertained comprehension of the question and response processes. Specifically, patients were asked to (1) restate each item in their own words; (2) describe how they arrived at their answer; (3) define words/phrases within particular items; (4) indicate whether the meaning of the item was clear to them; (5) indicate how confident they were about their answer when responding to the item; and (6) indicate whether the question was relevant to their experience with cancer. Each of the studies took this approach to investigate patients’ comprehension and response process for GP5. In addition, Study 1 featured additional targeted probes to elicit patient interpretations of the term bother, which features in GP5.

### Cognitive interview questions

The cognitive interviews in each study featured some common questions, including open and structured questions, as well as questions with a hybrid format. Open ended questions had no pre-specified response options and were used to elicit a descriptive, qualitative response from the patient. Close-ended questions had a pre-specified set of response options. Hybrid questions had a pre-specified set of response options as well as the opportunity for patients to expand on the responses in their own words. Table 1 shows the core set of 9 cognitive interview questions administered in each study and whether the question was administered as an open-ended, close-ended, or hybrid format question. These questions addressed how patients came to their response to GP5, the understandability and relevance of GP5, and the period of time patients thought about when answering GP5. The cognitive interview in Study 1 featured 8 additional targeted questions and probes on the term bother, which are also shown in Table 1. These additional questions explored what causes bother and how bother tracks with side effect severity.

**Table 1:** Cognitive interview questions

	Question administered as open (O), closed (C), or hybrid (H)		
Question	Study 1	Study 2	Study 3
*Core set of questions administered in Studies 1–3*			
What kinds of things did you think about when you answered the question? (i.e., How did you come to the answer you gave)?	O	O	O
How would you state the question in your own words?	O	O	O
Was the meaning of the question clear to you?^a^	H	H	
When responding, how confident did you feel about your answer to this question?^b^	C	C	C
Is this question relevant to your experiences with ≪ your cancer ≫ ?^c^	H	H	H
Is this relevant to your experience with your treatment and treatment side effects?^d^	C		
What does “bothered by side effects of treatment” mean to you (i.e., How do you define…)?	O		O
Do you have any questions about how to answer this question? Is everything clear and understandable?		H	
What period of time did you think about when you answered this question?			O
*Bother-specific questions and probes in Study 1*			
What is it about your side effects that bothers you?	O		
Imagine your side effects getting worse. Would you be more bothered by it?^d^	C		
How would it have to worsen to bother you?	O		
Imagine your side effects getting better. Would you be less bothered by it?^d^	C		
How would it have to improve to bother you less?	O		
Is the severity of treatment side effects something that determines how much it bothers you?^d^	C		
If you had severe side effects would it bother you more than if you have moderate treatment side effects?^d^	C		
Would moderate treatment side effects bother you more than mild treatment side effects?^d^	C		

^a^The hybrid format for these questions requested an initial response of “Yes” or “No.” Then, if the patient answered “No,” they were asked an open ended question asking to elaborate

^b^When administered as a close-ended question, response options were “Very confident,” “Confident,” and “Not at all confident”

^c^The specific cancer type relevant to the patient was asked about, i.e., Study 1: ovarian cancer, Study 2: non-Hodgkin’s lymphoma, and Study 3: colorectal or lung cancer

^d^Response options for these questions were “Yes” or “No”

### Data analysis

The data from Studies 1 and 2 were combined for analysis where possible since studies both included patients on treatment. Data were not combined for questions that were asked in only one of the studies. Since it included only treatment-naïve patients, Study 3 was analyzed separately. Participants’ demographic and clinical characteristics were summarized by study. These characteristics included age, gender, ethnicity, race, education level, ECOG PSR, cancer diagnosis, time since diagnosis, and whether the patient was currently on treatment or had previous cytoreductive surgery. Categorical variables were summarized with percentages and proportions, and continuous variables were summarized using means, standard deviations (SD), and ranges. Participants’ responses to structured questions were analyzed similarly, with summaries of responses shown for the overall sample and by study. De-identified responses to open-ended interview questions were first transcribed to text and then entered into a spreadsheet in Microsoft Excel for analysis. Responses to open-ended questions were analyzed via a constant comparative approach by one study team member with qualitative and measure development experience (J. D. P.) [24], wherein responses to open-ended questions from the first 5 interviews were reviewed to inform development of an initial codebook. The codebook was revised iteratively throughout analysis of subsequent participant responses to capture new codes, revise existing codes, and edit the codebook. After all participants’ data were coded in Excel, we reviewed all coded responses with the final codebook and made revisions to reflect updated codes, as needed. Next, we collapsed similar codes into broader themes. This approach is commonly applied to interpret qualitative data generated for the purpose of developing patient-reported outcome measures [25, 26]. Finally, the frequencies of responses falling under each theme were totaled.

## Results

### Sample characteristics

In Study 1, between February and April 2017, 282 patients were screened for eligibility. Of these, 41 were eligible. Of these, 21 consented to participant and completed a cognitive interview. In Study 2, between May and December 2015, 133 patients were screened for eligibility. Of these, 22 were eligible, and 19 consented to participate, with 15 completing a cognitive interview and 14 completing questions relevant to GP5. In Study 3, 26 patients were approached to participate. Sixteen patients consented and completed a cognitive interview. For Study 1, saturation was not used to guide recruitment. Participating patients’ demographic and clinical characteristics are shown in Table 2. Across the studies, patients were generally similar in terms of age and ethnicity, with a somewhat greater proportion of Black/African American patients participating in Study 3. Since Study 1 focused on ovarian cancers, all participants were female. Larger proportions of patients in Studies 2 and 3 attained college or advanced degrees and had an ECOG PSR value of 0.

**Table 2:** Selected demographic and clinical characteristics of study participants

	Study 1 (*N* = 21)	Study 2 (*N* = 14)	Study 3 (*N* = 16)
Age, mean (SD, range)	60 (9, 40–72)	66 (8, 49–81)	62 (10, 50–77)
Gender, % (*n*)			
Female	100% (21)	50% (7)	50% (8)
Male	0% (0)	50% (7)	50% (8)
Ethnicity, % (*n*)			
Hispanic/Latinx Origin	10% (2)	14% (2)	13% (2)
Not Hispanic/Latinx Origin	90% (19)	86% (12)	88% (14)
Race, % (*n*)			
White	76% (16)	86% (12)	69% (9)
African American/Black	14% (3)	7% (1)	23% (3)
Asian	5% (1)	0% (0)	0% (0)
Other	5% (1)	7% (1)	8% (1)
Education, % (*n*)			
8th grade or less	5% (1)	0% (0)	0% (0)
High school/GED	33% (7)	21% (3)	6% (1)
Some college/technical degree	48% (10)	14% (2)	25% (4)
College degree	10% (2)	36% (5)	31% (5)
Advanced degree	5% (1)	29% (4)	38% (6)
ECOG performance status, % (*n*)			
0	10% (2)	57% (8)	38% (6)
1	52% (11)	29% (4)	31% (5)
2	38% (8)	14% (2)	13% (2)
3	0% (0)	0% (0)	19% (3)
Cancer diagnosis			
Ovarian	62% (13)	-	-
Primary peritoneal	19% (4)	-	-
Ovarian and fallopian tube	14% (3)	-	-
Fallopian tube	5% (1)	-	-
Follicular lymphoma	-	64% (9)	-
Marginal zone lymphoma		29% (4)	-
Lymphoplasmacytoid lymphoma/Waldenström macroglobulinemia		7% (1)	-
Colorectal	-	-	69% (11)
Lung	-	-	31% (5)
Time since diagnosis, mean (SD, range)	1 year (2, 0–9)	8 years (5, 2–16)	1.5 months (1.2, 0–3.5)
Currently on treatment, % (*n*)	71% (15)	57% (8)	0% (0)
Cytoreductive surgery, % (*n*)	62% (13)	-	81% (13)

### GP5 understandability and relevance to patients on treatment

Responses to structured questions assessing GP5’s understandability and relevance to cancer patients provided by patients on treatment (Studies 1 and 2) are shown in Table 3. The overwhelming majority of patients (overall = 88%) reported that they would not rephrase GP5; the wording was acceptable. Similarly, the overwhelming majority reported that the meaning of GP5 was clear (overall = 97%). All but one patient reported being Very Confident or Confident in their ability to answer GP5. Similarly, all but one patient reported that GP5 was relevant to their experiences with cancer. In addition, all patients who answered GP5 reported that the question was relevant to their cancer treatment and treatment side effects.

**Table 3:** Understandability and relevance of GP5 in patients on treatment (Studies 1 and 2)

	Overall (*N* = 35)	Study 1 (*N* = 21)	Study 2 (*N* = 14)
How would you state this question in your own words?			
OK as is	88% (30)	81% (17)	100% (13)
Would rephrase	12% (4)	20% (4)	0% (0)
Was the meaning of the question clear to you?			
Yes	97% (33)	100% (21)	92% (12)
No	3% (1)	0% (0)	8% (1)
How confident were you in your ability to answer this question?			
Very confident	74% (25)	71% (15)	77% (10)
Confident	23% (8)	29% (6)	15% (2)
Not at all confident	3% (1)	0% (0)	8% (1)
Is this question relevant to your experiences with cancer?			
Yes	97% (34)	95% (20)	100% (14)
No	3% (1)	5% (1)	0% (0)
Is this relevant to your experience with your treatment and treatment side effects?^a^			
Yes	-	100% (9)	-
No	-	0% (0)	-

^a^This question was added to the Study 1 cognitive interview after several interviews were already completed

### How patients on treatment came to their response to GP5

**Fig. 1:**
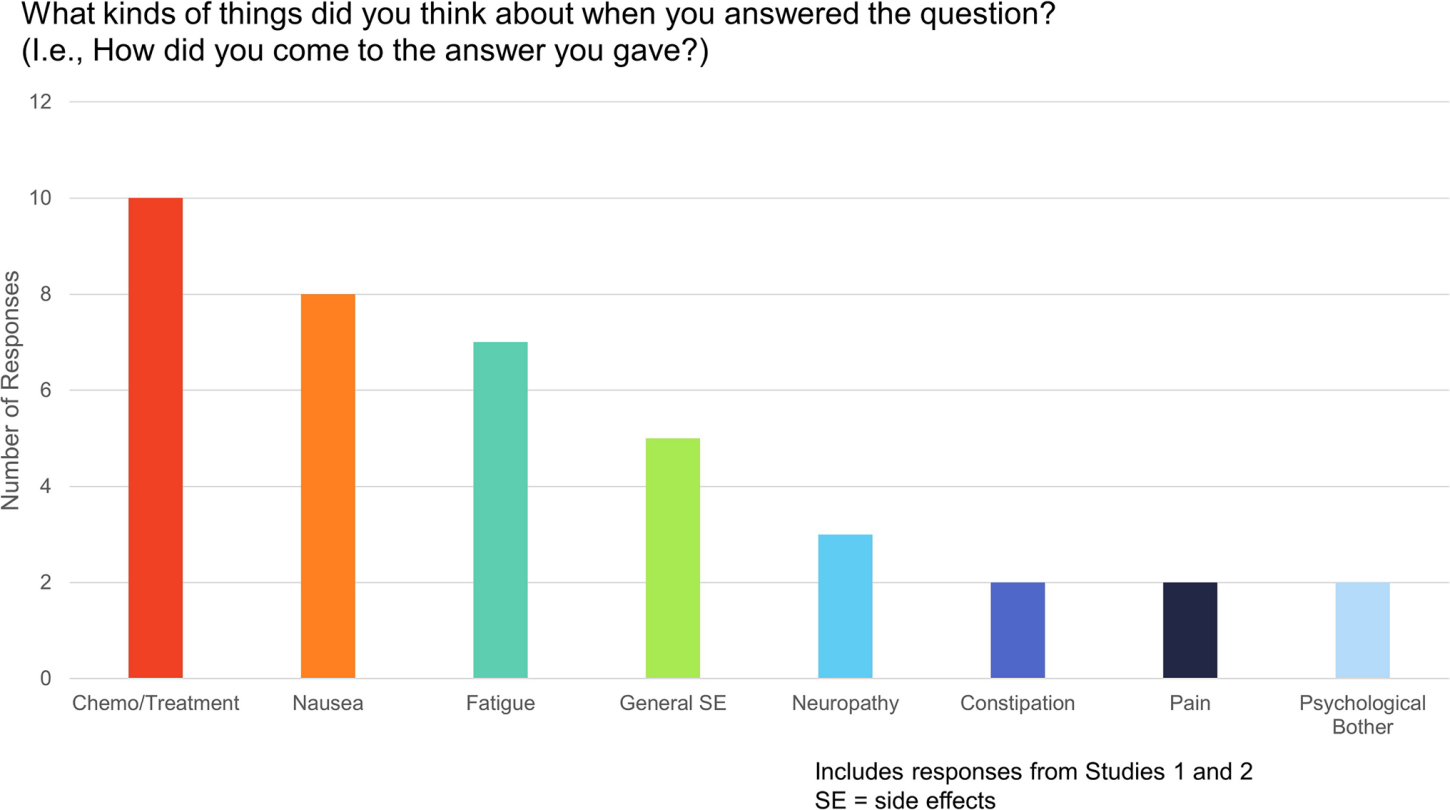
Themes emerging from responses to open-ended question on how patients come to their GP5 response

Analysis of responses to the open-ended question referring to GP5, “What kinds of things did you think about when you answered the question? (i.e., How did you come to the answer you gave?)” from patients in both Studies 1 and 2 yielded the following themes: chemotherapy/treatment, nausea, fatigue, general side effects, neuropathy, constipation, pain, and psychological bother (Fig. 1). Selected quotes from the qualitative analysis of additional open-ended questions in Study 1 (patients on treatment) targeted toward patients’ interpretation of the term bother are shown in Table 4. For the question, “What is it about your side effects that bothers you?,” the following themes emerged: specific side effects (e.g., “It’s the nausea that really bothers me. Fatigue doesn’t bother me as much…”), life/routine disruption (e.g., “Anything that is changing my routine.”), and psychological impact (e.g., “It’s psychological concerns and wondering if they are going to go away.”) For the question, “How would it have to worsen to bother you?,” the following themes emerged: more frequent treatment side effects (e.g., “If I had nausea every day. If I had no energy every day.”), more severe treatment side effects (“It would have to be more severe.”), reduced function/ increased dependency (“If I couldn’t get out of bed.”), and specific side effect worsened (e.g., “If I had less energy or worse nausea. […]”). For the question, “How would it have to improve to bother you less?,” the following themes emerged: side effects go away completely (e.g., “If the neuropathy would go away.”) and side effect severity reduces (e.g., “If I had minimal rather major lack of energy. If I had only a little nausea.”).

**Table 4:** Selected quoted responses to open-ended probes on the term “bother” (Study 1 only)

Question	Theme	Quote
What is it about your side effects that bothers you?	Specific side effect	• “It’s the nausea that really bother me. Fatigue doesn’t bother me as much. It’s difficult to eat and difficult to keep a positive attitude.” • “No one likes to feel crappy. It’s the nausea and vomiting.” • “I was thinking about the fatigue and lack of energy.” • “It’s the pain, neuropathy, and discomfort.” • “It’s the nausea and constipation.” • “It’s the pain in my hands and feet and the brain fog. It bothers me not being able to see the newspaper well when I am reading it.” • “[…] Lack of energy and fatigue.” • “The nausea, not feeling well. The hair loss and wearing a hat all the time.” • “It is the nausea and lack of energy. For the most part, I don’t have side effects.”
	Life/routine disruption	• “Anything that is changing my routine.” • “It’s the dependency, not being able to work and the lack of control.” • “It’s not doing the things you need to do at full [*sic*] throttle.” • “Not being able to run. And having a lack of energy.”
	Psychological impact	• “It’s psychological concerns and wondering if they are going to go away.” • “I am worried about trapped by feeling crappy.”
How would it have to worsen to bother you?	More frequent side effects	• “If I had nausea every day. If I had no energy every day.” • “It would have to be daily, more severe.”^a^
	More severe side effects	• “It would have to be daily, more severe.”^a^ • “It would have to be more severe.” • “It would have to worsen quite a bit.” • “They would have to be to the 10th degree or off the charts. […]”
	Reduced function/increased dependency	• “If it got as bad as it was 6 months ago when I couldn’t do anything — couldn’t get out of bed, couldn’t do anything on my own.” • “They would be more disruptive.” • “If I couldn’t get out of bed.” • “If I was more dependent or if I had to permanently retire.”
	Specific side effect worsened	• “I would be bothered by vomiting and if I felt worse that it meant my cancer is spreading.” • “If I couldn’t walk; if the pain in my hands and feet” • “If I couldn’t get rid of the thrush or the UTI didn’t go away.” • “If I had less energy or worse nausea. […]” • “If the neuropathy spread further in my hands and feet or if I had floppy feet and I had to use a wheel chair. If my vision got worse.”
How would it have to improve to bother you less?	Side effects go away completely	• “It would have to not be present.” • “If the neuropathy would go away.” • “Not being on the medication. If my hair grew back faster and I got my confidence back.” • “The UTI would go away. The constipation could not spasm.”
	Side effect severity reduces	• “If I had minimal rather major lack of energy. If I had only a little nausea.” • “If I could move around like I used to.” • “If the nausea improved.” • “It would be less pain full or if I could see better. If my headaches weren’t every day.” • “If I had energy.” • “Fewer instances, lower intensity or having better coping mechanisms.” • “If I feel better. Over the past few weeks, I have been feeling better.” • “If the fatigue would lessen. The pain and anxiety would have to improve.” • “If I had more strength, if I can start a project and don’t to sit down or take breaks.”

^a^Since this was a compound answer expressing two ways a symptom could worsen, it was used to support two themes

### Probes on the term “bother”

**Fig. 2:**
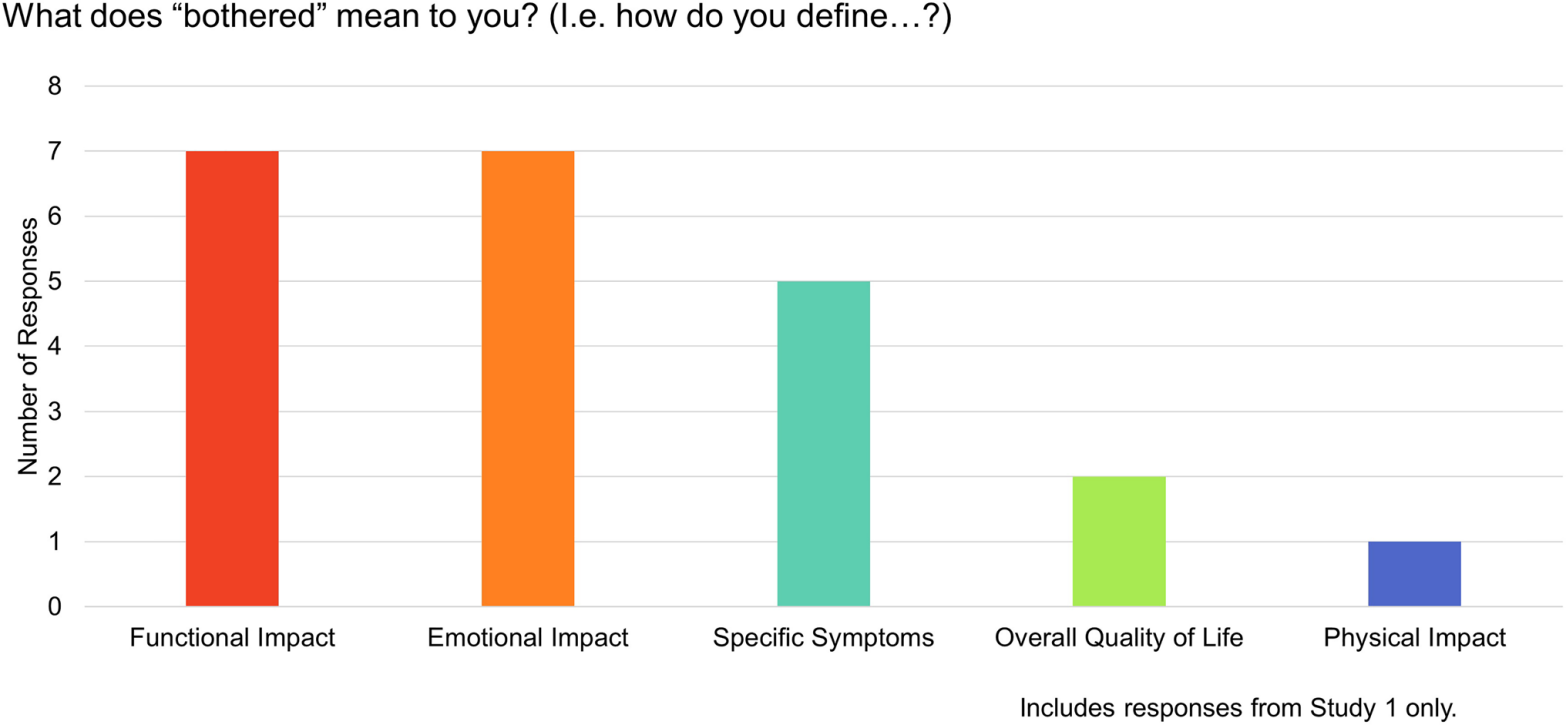
Themes emerging from responses to open-ended question on patients’ perspectives on the term ‘bother’

Structured questions from Study 1 about interpretation of the term bother demonstrated that, for a large majority of patients, the severity of side effects determines bother (Table 5). Moreover, our qualitative coding revealed that participants reported the expected relationship of bother to side effect severity wherein severe side effects would be more bothersome than moderate side effects, and moderate side effects would be more bothersome than mild side effects. An analysis of responses to the open-ended question, “What does ‘bothered’ mean to you? (i.e., how do you define…?),” among patients from Study 1 yielded the following themes: functional impact, emotional impact, specific side effects, overall quality of life, and physical impact (Fig. 2).

**Table 5:** Cognitive interview questions on the term “bother” (Study 1 only)

	*N* = 21
Imagine your side effects getting worse. Would you be more bothered by it?	
Yes	100% (17)
No	0% (0)
Imagine your side effects getting better. Would you be less bothered by it?	
Yes	88% (15)
No	12% (2)
Is the severity of treatment side effects something that determines how much it bothers you?	
Yes	81% (17)
No	19% (4)
If you had severe side effects would it bother you more than if you have moderate treatment side effects?	
Yes	95% (19)
No	5% (1)
Would moderate treatment side effects bother you more than mild treatment side effects?	
Yes	95% (19)
No	5% (1)
Would you answer differently to “I have side effects of treatment”?	
Yes	55% (45)
No	45% (9)

### GP5 understandability and relevance to treatment-naïve patients

Responses to both structured and open-ended questions from Study 3 about GP5’s understandability and relevance to treatment-naïve patients, along with how they came to their GP5 response, are shown in Table 6. A majority (57%) said GP5 was ok as is (would not rephrase) and 81% were either very confident or confident in their answer. Similar proportions said that GP5 was relevant (*n* = 7, 47%) and irrelevant (*n* = 6, 40%) to their cancer treatment, while 13% (*n* = 2) said they were unsure. Of the 40% who said GP5 was not relevant, half (*n* = 3) reported being “Not at all” bothered, two patients reported being “A little bit” bothered, and one could not decide between “Not at all” or “A little bit.” When asked what they thought of when they answered GP5, the largest proportion (*n* = 7, 44%) said they had negative expectations of side effects. Of these patients, four of the 7 reported being “Not at all” bothered on GP5, two reported being “A little bit” bothered, and one could not decide between “Not at all” or “A little bit.” Approximately one third (*n* = 5, 31%) said they could not answer and/or had no side effects. The remainder said they thought of surgery (*n* = 3, 19%) or specific side effects (*n* = 1, 6%). When asked what time period they thought of when answering GP5, patients responded variously with time spans ranging from over 4 weeks (*n* = 3, 20%) to right now (*n* = 2, 13%).

**Table 6:** Understandability, relevance, and what patients thought of when answering GP5 in treatment-naïve patients (Study 3)

	% (*n*)
How would you state this question in your own words?^a^	
OK as is	57% (8)
Would rephrase	43% (6)
How confident were you in your ability to answer this question?	
Very confident	56% (9)
Confident	25% (4)
Not at all confident	19% (3)
Is this question relevant to your cancer treatment?^b^	
Yes	47% (7)
No	40% (6)
Don’t know	13% (2)
What did you think about when you answered the question (i.e., How did you come to the answer you gave)?^c^	
No side effects/can’t answer	31% (5)
Surgery	19% (3)
Negative expectations about side effects	44% (7)
Specific side effects	6% (1)
What period of time did you think about when you answered this question?^b^	
Before past 4 weeks	20% (3)
Past 4 weeks	7% (1)
Past 1 week	20% (3)
Right now	13% (2)
No specific time	20% (3)
Unsure	20% (3)

With the exception of “How confident were you in your ability to answer this question?,” each question was open-ended. Categories for each question resulted from qualitative coding then frequencies responding within each category were calculated

^a^14 of 16 participants answered this question

^b^15 of 16 participants answered this question

^c^This was an open-ended question and patients’ responses were assigned to 1 of the 4 categories

## Discussion

Given increasing interest in the FACT GP5 as a patient-reported measure of tolerability in cancer trials, gaining additional insight into how patients determine how to respond to GP5 is important. We drew upon 3 qualitative studies totaling 51 patients of diverse cancer types. For patients currently on treatment, the results suggest that GP5 was highly comprehensible and relevant, and it captures critical aspects of the side effects of cancer therapies. In addition, the term bother did not compromise patients’ ability to respond to GP5, and this term appears to capture multiple aspects of side effect impact, including symptom severity and frequency as well as the functional impact of side effects. Although patients considered a variety of experiences when determining their responses to GP5, all of these considerations were germane to treatment tolerability. For treatment-naïve patients, there was more variation in GP5’s meaning and usefulness. This study supports the use of GP5 in its current form as a valid, brief measure of tolerability in cancer clinical research, especially for patients on treatment.

There was a close correspondence between patients’ reports of global bother and side effect severity as evidenced by the large proportions of patients who reported that bother would proportionally increase with side effect worsening, and vice versa (bother would proportionally decrease with side effect improvement). Responses also revealed that bother taps into side effect frequency (e.g., daily or persistent side effects) as well, although likely to a lesser extent. In addition, bother also seemed to capture elements of side effect impact, with multiple comments pointing to interference with daily activities or increased dependency. This diversity in interpretation of the term bother underscores GP5’s global nature and its flexibility to capture varying elements of side effect experience that different patients may find most important.

Responses about the comprehensibility of GP5 for patients on treatment were similar to those from a previous report by Jensen and colleagues [27] in a previous content validity study of the NFOSI-18 among ovarian cancer patients where 94% (17/18) of patients stated that they had no questions about how to answer GP5, 94% (16/17) stated that GP5 was understandable, and 88% (15/17) were very confident or confident in answering GP5. In the current study, over 90% of patients on treatment said the meaning of GP5 was clear and that they were very confident or confident in answering GP5 and 88% said they would not rephrase GP5. Taken together with these previous results, it appears that patients find GP5 understandable in its current form. In addition, similar to our study, Jensen and colleagues [27] found that patients commonly thought of fatigue, nausea, neuropathy, and vomiting when responding to GP5. Gastrointestinal side effects like nausea, vomiting, and constipation [28], as well as neuropathy [29], are among the most common and impactful chemotherapy side effects. Reports that patients commonly think of these side effects when responding to GP5 support the item’s face validity.

One concern with GP5 has been about its usefulness and interpretability among treatment-naïve patients or at baseline in trials. Although patients are slightly more likely to skip GP5 at baseline compared to other FACT-G items (5–15% skip GP5 vs an average 2–3% on other items), the vast majority of patients do respond to the item at baseline when administered in a trial, and nearly 10% report high side effect bother before any treatment starts [30]. However, what determines patients’ responses to GP5 before starting treatment has previously been unclear. Our study found that treatment-naïve patients are influenced by negative expectations or fears about potential side effects. Negative expectations about chemotherapy side effects have been reported elsewhere [31, 32]. In addition, it is notable that some treatment-naïve patients in Study 3 reported it would be challenging to answer GP5 or reported that it was not relevant to them prior to starting treatment. On the other hand, we note that all but one patient could give a response to the item when asked.

The potential for varying meanings for GP5 at baseline or lack of perceived relevance of this item at baseline may have implications for how to analyze GP5 in clinical trials. Depending upon the factors that contribute to the item response, change in GP5 scores from baseline may be difficult to interpret. In treatment-naïve patients at baseline, GP5 item response has, in one study, provided information about the future (post-baseline) GP5 values and treatment discontinuation [16]. In addition, a prospective cohort study of hormone-receptor-positive breast cancer patients who had recent surgery found a significantly higher relative risk (1.83; 95% CI: 1.03–3.26) for experiencing side effects 2 years post-baseline among patients who had high negative side effect expectations at baseline in comparison to those who had low negative side effect expectations at baseline [33]. The possibility that bother with other recent treatments (in this case surgery), or bother with side effects from other prior treatments, or negative expectations regarding pending treatment, could forecast subsequent tolerability is an area that could benefit from further research.

This study had both advantages and limitations. Advantages include the opportunity to elicit rich input through qualitative, individual interviews from patients with several different types of cancer. As PROs play a larger role in drug development and evaluation, emphasis has been placed by the US FDA on qualitative research to help determine whether these PROs capture the intended concepts and in ways that reflect patients’ experiences [34]. The data from this study will help support the use of GP5 in future cancer trials. On the other hand, like all qualitative studies, our results may not be generalizable to broader cancer populations. Additional research is needed to confirm and expand upon our findings. In particular, further qualitative work should examine what drives patients to select specific responses to GP5 over others in order to further elucidate the cognitive process involved in answering this item. An additional, methodological limitation was the use of only one coder to generate themes in our qualitative analysis.

In conclusion, we report patients’ perspectives on GP5’s understandability, including qualitative responses to open-ended questions. Our results indicate that GP5 is well-understood by patients, relevant to their experience with side effects, and able to flexibly capture several important elements of tolerability. This study suggests that GP5 is a useful, succinct measure that captures how cancer patients tolerate their treatment.

## Declarations

### Precis

Patient-reported approaches to capturing treatment tolerability in cancer clinical trials are needed. The FACT GP5 item, “I am bothered by side effects of treatment,” is an understandable, relevant, flexible way to capture overall side effect impact efficiently.
