# Correction to: How do patients interpret and respond to a single‑item global indicator of cancer treatment tolerability?

**DOI:** 10.1007/s00520-023-07953-7

**Published:** 2023-07-24

**Authors:** John Devin Peipert, Sara Shaunfield, Karen Kaiser, Patricia I. Moreno, Rina S. Fox, Sheetal Kircher, Nisha Mohindra, Edward Ip, Fengmin Zhao, Lynne Wagner, David Cella

**Affiliations:** 1 Department of Medical Social Sciences, Northwestern University Feinberg School of Medicine, 625 Michigan Ave, 21st Floor, Chicago, IL 60611 USA; 2 Department of Public Health Sciences, University of Miami Miller School of Medicine, Miami, FL USA; 3 University of Arizona College of Nursing, Tucson, AZ USA; 4 University of Arizona Cancer Center, Tucson, AZ USA; 5 Division of Hematology and Oncology, Department of Medicine, Northwestern University Feinberg School of Medicine and the Robert H. Lurie Comprehensive Cancer Center, Chicago, IL USA; 6 Department of Biostatistics and Data Science, Wake Forest School of Medicine, Winston-Salem, NC USA; 7 ECOG-ACRIN Biostatistics Center, Dana-Farber Cancer Institute, Boston, MA USA; 8 Department of Biostatistics, Harvard T. H. Chan School of Public Health, Boston, MA USA; 9 Department of Social Sciences and Health Policy, Wake Forest School of Medicine, Winston-Salem, NC USA

**Correction to: Supportive Care in Cancer (2023) 31:37**

**https://doi.org/10.1007/s00520-022-07484-7**

The article “How do patients interpret and respond to a single‑item global indicator of cancer treatment tolerability?”, written by Peipert, J.D., Shaunfield, S., Kaiser, K., Moreno, P.I., Fox, R.S., Kircher, S., Mohindra, N., Ip, E., Zhao, F., Wagner, L., Cella, D., was originally published Online First without Open Access. After publication in volume 31, issue 1, page 31:37 the author decided to opt for Open Choice and to make the article an Open Access publication. Therefore, the copyright of the article has been changed to © The Author(s) 2020 and the article is forthwith distributed under the terms of the Creative Commons Attribution 4.0 International License, which permits use, sharing, adaptation, distribution and reproduction in any medium or format, as long as you give appropriate credit to the original author(s) and the source, provide a link to the Creative Commons licence, and indicate if changes were made. The images or other third party material in this article are included in the article’s Creative Commons licence, unless indicated otherwise in a credit line to the material. If material is not included in the article’s Creative Commons licence and your intended use is not permitted by statutory regulation or exceeds the permitted use, you will need to obtain permission directly from the copyright holder. To view a copy of this licence, visit http://creativecommons.org/licenses/by/4.0.

The original article is corrected.

